# The Japanese version of the barcelona music reward questionnaire (J-BMRQ) confirms the cross-cultural generalizability of the “five-factor” model

**DOI:** 10.1371/journal.pone.0340700

**Published:** 2026-02-24

**Authors:** Shiori Honda, Ernest Mas-Herrero, Mina Isoda, Mai Muraki, Urbano Lorenzo-Seva, Yoichi Kitayama, Patrick E. Savage, Shinya Fujii

**Affiliations:** 1 Department of Psychiatry, Keio University School of Medicine, Tokyo, Japan; 2 Department of Cognition, Development and Educational Psychology, Institute of Neurosciences, University of Barcelona, Barcelona, Spain; 3 Cognition and Brain Plasticity Unit, Institut d’Investigacions Biomèdiques de Bellvitge (IDIBELL), Hospital Duran i Reynals, Hospitalet de Llobregat, Spain; 4 Japan Broadcasting Corporation, Tokyo, Japan; 5 Department of Psychology, Universitat Rovira i Virgili, Tarragona, Spain; 6 Faculty of Environment and Information Studies, Keio University, Kanagawa, Japan; 7 School of Psychology, University of Auckland, Auckland, New Zealand; Universidad Nacional de Tres de Febrero, ARGENTINA

## Abstract

The Barcelona Music Reward Questionnaire (BMRQ) is an instrument designed to assess individual differences in music pleasure and reward. While the BMRQ was developed in Spanish and English, it has recently been translated into French and Chinese. A point of debate is whether the five-factor model, initially proposed in the English and Spanish versions of the BMRQ, was consistently replicated in the translated versions. While the French version successfully reproduced the five-factor model, the Chinese version did not, potentially due to cultural variations. In this study, we developed a Japanese version of the BMRQ (J-BMRQ) and tested whether the J-BMRQ could replicate the five-factor model. Data from 1550 Japanese participants were collected and analyzed using the J-BMRQ, and its construct validity and reliability were tested via factor analyses. Our analyses supported the applicability of the five-factor model for the Japanese version of the BMRQ, demonstrating psychometric properties consistent with the original Spanish/English and French versions. Additionally, mirroring the earlier versions, a single-factor solution was informative for computing an overall BMRQ score. This study reinforces the cross-cultural generalizability of the BMRQ and offers a robust tool for future research on musical pleasure and reward in a Japanese context.

## Introduction

Individual differences constitute a fundamental topic within psychology and neuroscience, providing critical implications for understanding human personality, cognition, and behaviors [1,2]. Developing a tool for assessing these individual differences is paramount for researchers to investigate the unique traits that differentiate one person from another.

One domain where the study of individual differences proves significant is the realm of musical pleasure and reward processing [3]. Music, universal and diverse across human cultures, plays a pivotal role in shaping our personality, cognition, and behaviors [4,5]. To further comprehend the universality and diversity of human musicality, it is essential to assess individual differences in musical pleasure and reward processing.

The Barcelona Music Reward Questionnaire (BMRQ) was developed to assess individual differences in these areas [3]. However, despite its potential for widespread use, the BMRQ has been developed and translated into a limited set of languages, including Spanish/English [3], French [6], Chinese [7], and Danish [8]. By translating the BMRQ into additional languages and confirming its validity, we can investigate individual differences in musical pleasure and reward processing across a diverse range of linguistic and cultural backgrounds.

In the preceding study conducted by Saliba et al. (2016), the validity of the French version of the BMRQ was evaluated using factor analyses [6]. The original BMRQ consists of twenty items in total, with factor analysis revealing five factors: 1) Musical Seeking, 2) Emotion Evocation, 3) Mood Regulation, 4) Sensory-Motor, and 5) Social Reward. These five factors delineate distinct aspects of individual musical pleasure and reward experiences. Saliba et al. (2016) demonstrated that these five factors were comparably observed in the French version of the BMRQ, aligning with the original English and Spanish versions.

In another study conducted by Wang et al. (2021), the validity of the Chinese version of the BMRQ was also tested using factor analyses. Interestingly, the results derived from the Chinese BMRQ revealed a different factorial structure [7]. While the French version of the BMRQ aligned with the five-factor model consistent with the English and Spanish versions, the Chinese version did not. The results from the Chinese BMRQ were compatible with a revised model that excluded items 5 and 10 related to “dancing (e.g., music often makes me dance).” Specifically, the Sensory-Motor factor did not contribute to the outcomes of the Chinese BMRQ. This discrepancy could be attributed to cultural differences. Wang et al. (2021) suggested that China’s collectivist culture contrasts with Western countries that may foster individualistic cultures, and argue that dancing to music is a self-enhancing activity [9] and thus may be more prevalent in individualistic cultures.

In a recent validation study of the Danish version of the BMRQ, Lippolis et al. (2025) reported a factorial structure that diverged from the original model [8]. Specifically, the Sensory–Motor factor separated into two subfactors, one reflecting choreographed or performance-oriented dance and the other representing spontaneous synchronization or “groove,” potentially reflecting conceptual and cultural distinctions between planned artistic movement and casual music-induced movement. The Social Reward factor also split into two subfactors, one associated with interpersonal bonding through musical sharing and the other encompassing collective musical activities such as concerts and ensemble performance, along with the item related to financial cost. The authors suggested that these distinctions may be influenced by Denmark’s strong traditions in dance and performing arts, as well as cultural concepts such as *hygge*, which emphasizes intimacy, conviviality, and shared musical experiences.

A central question posed here is whether the Japanese version of the BMRQ can replicate the five-factor model consistent with the English and Spanish versions. If the patterns of musical pleasure and reward processing in the Japanese population were akin to those in Western countries, the results from the Japanese BMRQ would echo the five-factor model consistent with the English and Spanish versions. Conversely, if the musical pleasure and reward processing in the Japanese population paralleled those in China, the results from the Japanese BMRQ would not align with the five-factor model, as observed in the study by Wang et al. (2021). To address this, we developed a Japanese version of the BMRQ (J-BMRQ) and aimed to test whether the J-BMRQ could replicate the five-factor model consistent with its English and Spanish counterparts. We gathered data from the Japanese population using the J-BMRQ and tested its construct validity and reliability using factor analyses.

## Methods

### Questionnaire translation

For the forward translations, we utilized three different methods using the original English version as the basis. Two of the translations were completed by separate translation companies, while the third was carried out by a team consisting of two linguists, a bilingual student, an English teacher, and researchers. Following this, all translations underwent backward translation, conducted by translation companies distinct from those who performed the forward translations. Researchers subsequently reviewed and selected items that accurately conveyed the original meaning. The finalized J-BMRQ is available on the Open Science Framework (https://osf.io/s3uwr/files/osfstorage).

### Data collection

We collected data via an online form provided by the Citizen, which is hosted by the Japan Broadcasting Corporation (Japanese name: NIPPON HOSO KYOKAI; NHK) from participants whose first language was Japanese and who were aged 18 years or older. The survey form was available at the following URL: https://www.nhk.or.jp/citizenlab/music_reward/index.html). Data was collected from March 15th, 2022, to August 26th, 2022. Before enrollment, participants were presented with NHK’s privacy policy and a detailed explanation of the study. They were required to provide written informed consent by selecting an agreement checkbox before proceeding with the survey. Participants who did not provide consent were unable to proceed further. For minors, the consent document was read together with their guardians, and participation was allowed only if guardian consent was obtained. The Research Ethics Committee of Keio University Shonan Fujisawa Campus approved the experimental protocol (approval number: 396).

Participants answered the J-BMRQ and provided demographic information (age, gender, first language, and music experiences). A total of 1579 participants were initially included in this study. Eight participants were excluded as they did not complete the J-BMRQ. Another eight participants were removed from the dataset due to insufficient information regarding their proficiency in Japanese. An additional 13 participants were excluded because they had previously completed the BMRQ. Consequently, a total of 1550 data were used in the present study (Table 1). The mean age of participants was 36.34 ± 16.9 years (mean ± standard deviation: SD). Of the total sample, 1060 participants were female, constituting 68.39% of the sample, and 464 were male, constituting 30% of the sample. Eight hundred and eighty-eight participants (57.29% of the samples) had musical training, excluding music education received as part of compulsory schooling. The mean duration of musical training was 8.33 ± 5.57 years (mean ± SD). All participants held Japanese citizenship. Although 15 participants were not born in Japan, and 12 participants were not currently residing in the country, each prefecture was represented by at least 4 participants who were either born there or had lived there. Tokyo, the most populous city in Japan, had the highest participant representation in the present study with 18.77% of participants having been born there and 24.06% currently residing there.

**Table 1:** Demographic information.

Variables	
**Number of Participants**	1550
**Age in Years** (Mean ± Standard Deviation)	36.336 ± 16.896
**Number of Females**	1060
**Number of Participants** **with Musical Training Experience**	888
**Duration of Musical Training in Years** (Mean ± Standard Deviation)	9.362 ± 7.187

### Data analysis

The BMRQ comprises twenty items in total, each measured on a five-point scale ranging from “fully disagree” [1] to “fully agree” [5]. We first calculated the means and standard deviations (SDs) for each item in the dataset. We also computed the overall score by summing the raw scores of all items, except for items 2 and 5, which needed to be reverse-scored [3]. Given that each of the twenty item is scored on five-point scale, the theoretical range for the overall score extends from 20 (1 point × 20 items) to 100 (5 points × 20 items). The means and SDs of each item, alongside the overall score of the J-BMRQ in this study, were compared with those of the original Spanish version of the BMRQ (Table 2).

**Table 2:** Item-item comparison between the original Spanish and Japanese versions of the BMRQ.

Item	Japanese	Spanish
**Q1**	4.02 ± 1.01	3.85 ± 0.86
**Q2**	1.73 ± 0.98	1.70 ± 0.92
**Q3**	4.17 ± 0.96	4.30 ± 0.79
**Q4**	4.16 ± 1.00	4.17 ± 0.91
**Q5**	2.37 ± 1.23	1.65 ± 1.02
**Q6**	4.30 ± 0.90	3.74 ± 0.86
**Q7**	4.14 ± 1.00	3.89 ± 0.98
**Q8**	4.23 ± 0.99	4.53 ± 0.70
**Q9**	4.41 ± 0.82	4.26 ± 0.82
**Q10**	3.44 ± 1.22	3.96 ± 1.03
**Q11**	3.16 ± 1.19	3.46 ± 0.86
**Q12**	3.76 ± 1.27	3.55 ± 1.06
**Q13**	3.95 ± 1.19	3.28 ± 1.25
**Q14**	4.35 ± 0.81	4.35 ± 0.78
**Q15**	4.54 ± 0.76	4.29 ± 0.76
**Q16**	4.30 ± 1.00	3.82 ± 0.92
**Q17**	3.44 ± 1.34	2.29 ± 1.12
**Q18**	4.33 ± 0.95	3.94 ± 0.88
**Q19**	4.30 ± 0.91	4.11 ± 0.98
**Q20**	4.42 ± 0.90	4.00 ± 0.91
**the overall raw score**	81.32 ± 11.16	78.42 ± 10.47

Note that the overall raw score was computed by summing the scores from all items, except for items 2 and 5, which needed to be reverse-scored.

We investigated the relationship between demographic information and the overall score of the J-BMRQ. We tested whether there were differences in the overall score of the J-BMRQ between males and females and between participants with and without musical training. Because the overall score data did not follow a normal distribution, we used the Mann-Whitney U test to compare males to females and to compare participants with and without musical training. Additionally, we performed correlation analyses using Spearman’s correlation coefficients to determine if the overall score of the J-BMRQ related to age or duration of music training. Spearman’s correlation was selected because normality assumptions were not met, as assessed using the Shapiro–Wilk test. We additionally examined correlations between age and BMRQ scores within three age groups: < 18 years, 18–59 years, and ≥60 years. Age groups were defined to reflect developmental stages and to enable comparison with previous studies [8,10]. The alpha level for all statistical tests was set at 0.05. The analyses were conducted using IBM SPSS Statistics Version 26 and R Version 4.4.1.

To validate and assess the replicability of the J-BMRQ, we performed a confirmatory factor analysis (CFA) and a cross-validation using FACTOR software [11]. For the CFA, a model of five correlated factors was proposed, following the rotated matrix identified in Mas-Herrero et al. [3]. Robust factor analysis was used to compute unweighted least square estimates from the polychoric correlation matrix, calculated for the 20 items of the translated questionnaire. A semi-specified orthogonal Procrustes rotation [12] was then carried out to estimate the factor loadings of each item on each of the five content factors.

To determine whether the correlation matrix was suited for factor analysis, we computed the Kaiser–Meyer–Olkin Test (KMO) for sampling adequacy [13]. We also examined the RMSEA, CFI, and GFI to assess the goodness of fit of the factor solution. Additionally, we inspected the congruence values [14] between the rotated loading matrix and the target loading matrix; a congruence value above.85 indicates a fair similarity.

Subsequently, we computed the ORION reliability index for each factor to estimate factor reliabilities [15]. The quality of the factor solution was further evaluated by inspecting the Factor Determinacy Index (FDI), Sensitivity Ratio (SR), and Expected Percentage of True Differences (EPTD) [16]. ORION reliabilities above 0.80, an FDI above 0.90, an SR above 2, and EPTDs above 90% indicate that factors are reliable and suitable for individual assessment.

Construct replicability was assessed using the H index, which evaluates the extent to which a set of items represents a common factor. High H values (> 0.80) suggest a well-defined latent variable that is likely to remain stable across studies [16]. We computed Cronbach’s alpha for the overall scale to assess internal consistency.

To further confirm the reproducibility of the identified structure, we conducted an Objectively Refined Target Matrix (RETAM) cross-validation [17]. To implement the cross-validation, the sample was first split into two random subsamples using SALOMON [18]. This method optimally divides the sample into two equivalent halves, ensuring the representativeness of the subsamples. Next, the RETAM procedure was applied to the first subsample to obtain a refined target matrix, which served as a fixed target matrix for the second subsample. If the rotated loading matrix in the second subsample aligns with the rotated loading matrix in the first subsample, we can confidently assert that the final solution fits not only the sample data but also the population data. As per Lorenzo-Seva & ten Berge (2006), a congruence value greater than.95 indicates that the factor solution has been fitted to the population, not merely to the sample at hand [19].

Although the 5-factor structure of BMRQ is reliable and presents acceptable psychometric quality indices, previous versions (including the original) indicate that a single dimension could also be interpreted. To assess the unidimensionality of the J-BMRQ, we inspected inter-factor corrections, unidimensional congruence (UNICO), explained common variance (ECV), and mean of item residual absolute loadings (MIREAL). Values of UNICO greater than 0.95, ECV greater than 0.85, or MIREAL less than 0.30 suggest that data can be treated as essentially unidimensional [16]. Finally, as the indices confirmed that a single dimension could be interpreted, we also inspected the unidimensional factor solution.

## Results

### Relationship between demographic information and overall score

A significant sex difference was observed in the overall scores of the J-BMRQ, with females scoring higher than males (*U* = 225493, *p* = 0.010, effect size *r* = −0.066; Males: mean ± SD = 80.164 ± 11.776, Females: mean ± SD = 81.880 ± 10.891). Participants with a background in music training scored higher (*n* = 888, mean ± SD = 81.956 ± 11.145) than those without such experience (*n* = 662, mean ± SD = 80.456 ± 11.138; *U* = 310052.5, *p* = 0.002, effect size *r* = 0.078). Correlation analyses showed no significant relationship between age and the overall scores of the J-BMRQ (*ρ* = −0.043, *p* = 0.090). Additionally, we found a significant correlation between the duration of music training and the BMRQ score, although the effect size was small (*ρ* = 0.091, *p* = 0.008). Additionally, in the analysis dividing participants into three age groups, age in the under-18 group showed a positive correlation with BMRQ total scores (n = 148; r = 0.266, p = 0.001). In the 18–59 group, the correlation was weakly negative (n = 1252; r = −0.100, p < 0.001), whereas in the over-60 group, no significant correlation was observed (n = 150; r = −0.154, p = 0.060).

### Confirmatory factor analysis

The inter-item polychoric correlation matrix was deemed suitable for factor analysis (KMO = 0.891), and the factor analysis solution reached acceptable goodness-of-fit levels (RMSEA = 0.044, CFI = 0.992, and GFI = 0.995).

The congruence values between the rotated and the ideal loading matrices ranged from 0.84 to 0.94 for the five factors, with an overall congruence of 0.88. Given that the coefficients were either above or close to the threshold of 0.85 (the Social Reward facet presented a congruence value of 0.84), the factor similarity between the rotated loading matrix and the ideal loading matrix can be regarded as fair [19].

ORION reliabilities of the five dimensions ranged from 0.82 to 0.92. The obtained ORION reliabilities, alongside the FDI (ranging from 0.91 to 0.96), SR (ranging from 2.15 to 3.28), and EPTD (ranging from 89.4 to 93.2) scores, indicate acceptable quality for the five dimensions. The questionnaire also exhibits good internal consistency (Cronbach’s alpha: 0.91).

We then evaluated the H indices to determine how well each factor was represented by its corresponding items. H-latent assesses how well the factor can be identified by the continuous latent response variables underlying the observed item scores (i.e., the factor score estimates). Conversely, H-observed assesses how well it can be identified from the observed item scores (i.e., the score obtained from summing raw participants’ responses). H-latent scores ranged from 0.82 to 0.92, while H-observed ranged from 0.74 to 0.82. Since all H-latent values were above 0.80 and consistently larger than the corresponding H-observed values, factor scores should be computed to estimate each factor instead of merely summing the response of the corresponding items of each factor. Table 3 shows the results of the factorial loading matrix. An Excel file that computes factor scores based on participant’s responses to the items is provided in the Supporting Information.

**Table 3:** Results of multidimensional factor analysis.

Item		MS	EE	MR	SM	SR
7	*I inform myself about music I like.*	**0.83**	0.02	-0.1	-0.1	0.09
11	*I’m always looking for new music.*	**0.57**	-0.1	0.05	0.15	-0.1
17	*I spend quite a bit of money on music and related items.*	**0.52**	0.17	-0.2	-0.1	0.26
2	*In my free time I hardly listen to music.*	**-0.5**	0.08	**-0.3**	-0.1	0.12
8	*I get emotional listening to certain pieces of music.*	-0	**0.83**	0.04	0.03	-0.1
12	*I can become tearful or cry when I listen to a melody that I like very much.*	-0.1	**0.74**	-0	0.01	0.05
18	*I sometimes feel chills when I hear a melody that I like.*	0.25	**0.59**	-0	0.01	-0
3	*I like to listen music that contains emotion.*	-0.1	**0.35**	0.22	-0	0.1
14	*Music helps me chill out.*	0.03	-0.1	**0.98**	-0	-0
9	*Music calms and relaxes me.*	-0	-0	**0.91**	-0	-0.1
4	*Music keeps me company when I’m alone.*	-0	-0	**0.56**	-0	0.14
19	*Music comforts me.*	0.03	**0.35**	**0.48**	-0	0.04
10	*Music often makes me dance.*	0.03	-0	0.02	**0.95**	-0.1
5	*I don’t like to dance, not even with music I like.*	0.04	0.07	0.1	**-0.9**	-0
20	*When I hear a tune I like a lot I can’t help tapping or moving to its beat.*	0.1	0.12	0	**0.6**	0.09
15	*I can’t help humming or singing along to music that I like.*	-0.1	0.09	0.21	**0.35**	0.19
6	*Music makes me bond with other people.*	0.05	-0.1	0.07	-0.1	**0.9**
1	*When I share music with someone I feel a special connection with that person.*	0.03	-0	0.17	-0.1	**0.57**
16	*At a concert I feel connected to the performers and the audience.*	-0.1	0.03	0.06	0.15	**0.55**
13	*I like to sing or play an instrument with other people.*	0.03	-0	0.02	0.13	**0.51**

Factorial loading matrix for each item for the multidimensional factor analysis (values larger than absolute 0.3 are in boldface). *MS = Music Seeking, EE = Emotion Evocation, MR = Mood Regulation, SM = Sensory-Motor, SR = Social Reward.*

### Cross-validation analysis

Congruence indices from the cross-validation analysis ranged from 0.95 to 0.98 for the five factors, with an overall congruence of 0.96. These congruence values, all above the 0.95 threshold, indicate that the factor solution can be fitted to the population.

### Unidimensionality assessment

Finally, inter-factor correlations ranged between 0.42 and 0.67 (Table 3). Furthermore, the indices for assessing unidimensionality were UNICO = 0.94, ECV = 0.81, and MIREAL = 0.22. These values indicate that the five factors were moderately correlated and that a single dimension could also be acceptable for the current dataset. Hence, a second factor analysis incorporating one factor was computed. Although the goodness-of-fit indices were slightly less optimal, they were still acceptable (RMSEA = 0.108, CFI = 0.90, and GFI > 0.999). The loading values within the single factor ranged from 0.44 to 0.73. The EAP reliability of the factor was 0.92. The H-latent and H-observed indices were 0.92 and 0.91, respectively. Considering that both indices are similar and considerably above 0.80, the overall BMRQ factor can be computed by simply summing participants’ responses to items.

**Fig 1:**
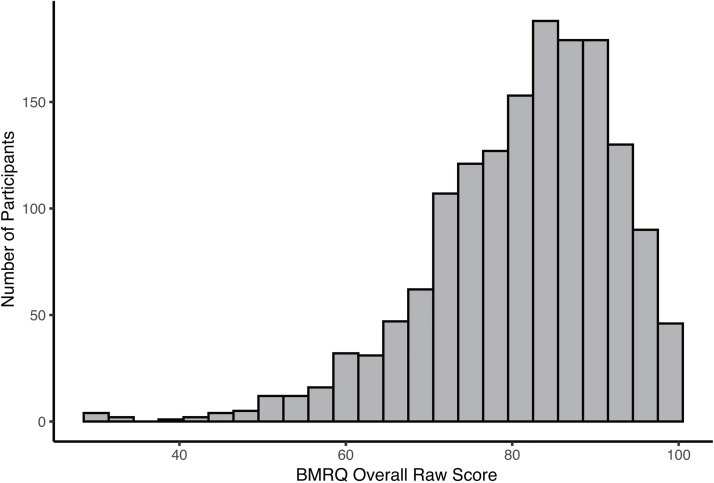
Histogram of the overall score computed in this study (n = 1550).

Overall, the analyses indicate that the five-factor model was suitable for the J-BMRQ, showing similar psychometric properties to the original and French versions. Additionally, in line with the previous versions, a single factor would also be valuable in computing an overall BMRQ score. The distribution of the overall scores (global sensitivity to music reward) in the J-BMRQ in this study was similar to the patterns observed in both the original and French versions (Fig 1).

## Discussion

The present study developed and validated a Japanese version of the Barcelona Music Reward Questionnaire (J-BMRQ), to test its consistency with the five-factor model observed in the English and Spanish versions of the BMRQ. Utilizing data from 1550 participants, our analyses revealed that the five-factor model was an acceptable fit for the J-BMRQ. This finding aligns with the psychometric properties identified in the original Spanish/English and French versions but contrasts with the results of Wang et al. (2021), which suggested discrepancies in the Chinese version of the BMRQ.

Previous studies on the BMRQ in Spanish, English, and French consistently reported similar factorial structures [3,6]. However, the Chinese version displayed a different pattern [7]. The results from the Chinese BMRQ aligned with a revised model that excluded items 5 and 10, both related to dancing—a subcategory of the Sensory-Motor domain. Wang et al. (2021) noted that dancing, closely associated with the social function of music, could be influenced by cultural values such as collectivism and individualism [7,20]. They also cited a study by Boer et al. (2012), which suggested that individuals in individualist cultures are more inclined to dance while listening to music compared to those from collectivist cultures [7,9]. This tendency can be attributed to the perception of dancing as a self-enhancing activity in individualistic societies [9]. Drawing on this evidence, Wang et al. (2021) posited that the inherent collectivism in Chinese culture might have influenced these results.

Similarly, the Danish version of the BMRQ also departed from the original factor structure. Lippolis et al. reported that the Sensory–Motor domain split into “Dance” (choreographed or performance-oriented movements) and “Spontaneous Synchronization” (unplanned bodily responses to rhythm), while the Social Reward domain separated into intimate interpersonal connections and collective musical activities. These differences were attributed to Denmark’s distinctive dance traditions, active participation in performing arts, and the cultural concept of *hygge* [8].

Conversely, our findings closely matched those from the Spanish, English, and French versions. If cultural values of collectivism and individualism affect this topic, we need to consider where Japanese culture fits in. Conventionally, the Japanese population is perceived as collectivistic, a commonly held view in psychology [21]. However, a previous study that examined psychological comparisons of collectivism and individualism between Japan and the U.S. found no significant differences in 16 out of the 22 studies [21]. Another study suggested that the extent of collectivism versus individualism in Japan may be regarded as intermediate between China and Western countries [9]. Taken together, the differences between geographically close countries such as Japan and China, and France and Denmark, indicate that factors beyond geographical proximity, such as domestic cultural contexts, can strongly shape how musical reward is perceived. Future research should therefore consider not only individual musical experiences but also broader cultural backgrounds when examining cross-cultural differences in the BMRQ.

However, a potential factor influencing the results is the age range of the samples. It’s important to highlight the age range differences between the Chinese samples from Wang et al. (2021) and the Japanese samples in this study. The Chinese samples from Wang et al. (2021) primarily consisted of younger participants, aged 16–23 years. On the other hand, our study encompassed a broader age range (7–82 years, mean ± SD: 36.34 ± 16.9), similar to the previous Spanish study (18–78 years, mean ± SD: 33.9 ± 10.0). This suggests that the age range may be a contributing factor to the discrepancies observed between the Chinese study and others. Wang et al. (2021) also suggested that their recruitment of a younger population might have influenced the results, as this demographic is often less confident in dancing [7,22]. Similarly, Lippolis et al.’s study was conducted in Danish teenagers. Given these age differences—and the fact that both the Chinese and Danish versions diverged specifically within the Sensory–Motor facets—an equally plausible explanation is developmental: the latent structure of the BMRQ, particularly dance-related indicators, may shift from adolescence into adulthood. This interpretation suggests age-related non-invariance, meaning the questionnaire may not measure the underlying constructs in the same way across age groups, which would make adolescent and adult scores not directly comparable. It therefore warrants explicit tests of developmental measurement invariance and, ideally, longitudinal designs to disentangle age from cohort effects before attributing discrepancies solely to culture.

In our study, we did not find a significant correlation between age and the overall BMRQ score. However, compared with the balanced age distribution in Belfi et al. (20–85 years, evenly sampled by decade), our sample had fewer older participants and included younger individuals, which may have contributed to the absence of a statistically significant negative correlation despite a similar trend. To address this point, we conducted an additional analysis by dividing participants into three clearly defined age groups: under 18, 18–59 years, and 60 years or older. The results showed a positive correlation between age and musical reward in the under-18 group, partly consistent with prior findings, a weak negative correlation in the middle-aged group, and no significant correlation in the over- 60 group. This result may reflect the predominance of participants in their 60s within this age category. Taken together, these findings suggest that the relationship between age and musical reward sensitivity across the lifespan may follow a non-linear pattern, characterized by a sharp increase before age 18 and a gradual decline thereafter. This trajectory resembles age-related patterns in the dopamine system, which plays a key role in musical reward processing (e.g.,[23]). Future research should examine musical reward while explicitly considering developmental and aging processes.

Gender distribution is another pivotal factor to consider. In Wang et al.’s study, females constituted a significant majority, making up 73.66% of the sample with 825 out of 1120 participants. In our study, females represented 68.39% of the sample, equating to 1060 out of 1550 participants. The Spanish study comprised 53% females from a total of 804 participants. The Chinese sample [7] had a proportionally larger female representation than the other studies, potentially influencing the results. Notably, prior research has shown that the Spanish female population scored higher on the BMRQ compared to males [3]. Consistently, we also found significantly higher BMRQ scores in females compared to males, suggesting a heightened sensitivity to musical reward among females. However, because the effect size in this study was small (effect size *r* = −0.06), we need further research to explore the gender differences in musical reward sensitivity. Also, future studies should consider how gender distribution affects the factorial structure.

Another factor to consider is musical training. In this study, 57.29% of participants (888 out of 1550) had musical training, with the average duration being 8.33 years. In the original Spanish version, 14% of the 808 participants living in Barcelona identified themselves as professional musicians whose primary occupation and source of income was music [3]. Additionally, 40% of the non-professional participants reported having musical studies [3]. In the French version of the BMRQ, musicians constituted 22.5% of the sample (231 out of 1027 participants) [6]. In contrast, in Wang et al.’s study, only about 10% of participants had musical training, with the average duration being 3.5 years. This suggests that participants involved in the Chinese version of the BMRQ might have less musical training than those in the other studies. In this study, we found that the participants with a background in music training scored higher than those without such experience. Previous study also found that BMRQ sub-scores in musicians are higher than those of non-musicians, indicating that music training experience is an important factor in music rewards [3]. Since musical training often involves sensory-motor skills necessary to play music, this could help explain why the Sensory-Motor factor did not fit the results from the Chinese BMRQ.

There are several limitations to our study. First, we were not able to examine regional differences within Japan. Although NHK is a nationwide broadcaster, it was difficult to recruit participants evenly from all regions. Yoshino et al. (2022) have reported that personality traits vary across regions within Japan, suggesting that future studies should increase the sample size and explicitly examine such regional differences [24]. Additionally, given that the present study was conducted via an online survey, the sample may have been biased toward individuals with higher internet literacy, particularly among older age groups. Moreover, although the online form was hosted on a dedicated site by the NHK, which is a nationally broadcast public service media organization in Japan, the sample may have included a relatively higher proportion of NHK viewers. Another limitation is that we did not account for whether participants were professional musicians. Musical training years were determined solely based on whether they had received instruction at a music school. In future studies, we will consider comparing BMRQ scores between professional and non-professional musicians. Thus, to more confidently assess cultural influences on the BMRQ structure, future work should employ harmonized cross-national designs with balanced, representative samples across regions and age groups. In this sense, our study provides a rigorously translated and psychometrically validated Japanese BMRQ based on a large, broadly aged sample, reinforcing the original five-factor model and establishing a solid baseline for future cross-cultural work. Finally, the original English version of the BMRQ was used as the source instrument in this study [3]. The extended version of the BMRQ (eBMRQ) was not adopted because data collection for the Japanese version was completed prior to the publication of the eBMRQ [25]. Future studies will be needed to examine the validity of a Japanese version of the eBMRQ.

In this sense, our study provides a rigorously translated and psychometrically validated Japanese BMRQ based on a large, broadly aged sample, reinforcing the original five-factor model. Therefore, the J-BMRQ may represent a solid baseline for future cross-cultural work and a means to improve our understanding of the psychological and neural mechanisms underlying individual differences in musical reward sensitivity [26].

## Conclusion

In conclusion, our analyses support the suitability of the five-factor model for the Japanese version of the BMRQ, reflecting psychometric properties consistent with the original Spanish/English and French versions. Furthermore, akin to the original version, a single-factor solution could be beneficial for calculating an overall BMRQ score. This study strengthens the cross-cultural generalizability of the BMRQ and provides a robust tool for future research on musical pleasure and reward processing in the Japanese context.

## Supporting information

S1 File Excel file to compute factor scores. This file includes a spreadsheet with embedded formulas to calculate the five factor scores of the BMRQ from participant responses.
(XLSM)

S2 File Original version of the BMRQ. This file contains the original English version of the BMRQ.
(XLSX)
